# SETMAR isoforms in glioblastoma: A matter of protein stability

**DOI:** 10.18632/oncotarget.14218

**Published:** 2016-12-25

**Authors:** Audrey Dussaussois-Montagne, Jérôme Jaillet, Laetitia Babin, Pierre Verrelle, Lucie Karayan-Tapon, Sylvaine Renault, Cécilia Rousselot-Denis, Ilyess Zemmoura, Corinne Augé-Gouillou

**Affiliations:** ^1^ EA 6306 IGC, University François Rabelais, 37200 Tours, France; ^2^ UMR CNRS 7292 GICC, University François Rabelais, 37000 Tours, France; ^3^ EA 7283 CREaT, Université d′Auvergne, BP 10448, 63000 Clermont-Ferrand, France; ^4^ Institut Curie, Dpt d'Oncologie Radiothérapique, 75005 Paris, France; ^5^ Centre Jean Perrin, Service Radiothérapie, Laboratoire de Radio-Oncologie Expérimentale, 63000 Clermont-Ferrand, France; ^6^ INSERM U1084, Laboratoire de Neurosciences Expérimentales et Cliniques, F-86021 Poitiers, France; ^7^ University of Poitiers, F-86022 Poitiers, France; ^8^ CHU of Poitiers, Laboratoire de Cancérologie Biologique, F-86021 Poitiers, France; ^9^ CHRU of Tours, Unité d’Anatomie et Cytologie Pathologiques, 37000 Tours, France; ^10^ INSERM U930 Imagerie & Cerveau, University François Rabelais, 37000 Tours, France; ^11^ CHRU of Tours, Service de Neurochirurgie, 37000 Tours, France

**Keywords:** glioblastoma, SETMAR, alternative ATG, protein half-live, NHEJ repair

## Abstract

Glioblastomas (GBMs) are the most frequent and the most aggressive brain tumors, known for their chemo- and radio-resistance, making them often incurable. We also know that SETMAR is a protein involved in chromatin dynamics and genome plasticity, of which overexpression confers chemo- and radio-resistance to some tumors. The relationships between SETMAR and GBM have never been explored. To fill this gap, we define the SETMAR status of 44 resected tumors and of GBM derived cells, at both the mRNA and the protein levels. We identify a new, small SETMAR protein (so called SETMAR-1200), enriched in GBMs and GBM stem cells as compared to the regular enzyme (SETMAR-2100). We show that SETMAR-1200 is able to increase DNA repair by non-homologous end-joining, albeit with a lower efficiency than the regular SETMAR protein. Interestingly, the regular/small ratio of SETMAR in GBM cells changes depending on cell type, providing evidence that SETMAR expression is regulated by alternative splicing. We also demonstrate that SETMAR expression can be regulated by the use of an alternative ATG. In conclusion, various SETMAR proteins can be synthesized in human GBM that may each have specific biophysical and/or biochemical properties and characteristics. Among them, the small SETMAR may play a role in GBMs biogenesis. On this basis, we would like to consider SETMAR-1200 as a new potential therapeutic target to investigate, in addition to the regular SETMAR protein already considered by others.

## INTRODUCTION

The chimeric enzyme SETMAR (or METNASE) has been discovered in the human genome twenty years ago [1] and its molecular evolution in anthropoid primates was achieved at the beginning of the 21st century [2, 3]. The SET domain is encoded by exons 1 and 2 of *SETMAR*, and the MAR domain by exon 3. Studies involving recombinant SETMAR expressed in cultured cells revealed multiple biochemical functions including Histone 3 methylation at Lys4 and Lys36 [3, 4], Lys130 methylation of splicing factor snRNP70 [5], chromosomal decatenation [6], and non-homologous end-joining (NHEJ) DNA repair [7]. Albeit recent data seemed indicate that the MAR domain of SETMAR was necessary for DNA repair [8, 9], it has never been demonstrated to be sufficient (when acting alone) for DNA repair. Briefly, SETMAR was demonstrated to be a partner for hPSO4 [10], CHK1 [11] and LIGASE IV [7] in double strand break repair by NHEJ. In addition, SETMAR can play a direct role in the joining of both compatible and non-compatible ends during NHEJ [8, 12], whereas the over-expression of SETMAR did not produce any significant changes in homologous recombination repair [3]. Finally, SETMAR was shown to be implicated in the repair of collapsed forks [13, 12]. SETMAR has also conserved many functions coming from the *Hsmar1* transposase like site specific DNA binding to the *Hsmar1* transposon ends, single strand DNA cleavage and DNA integration [14, 15]. In addition to its roles in chromatin dynamics and genome plasticity, a positive correlation was established between SETMAR overexpression and certain cancers (leukemia, breast cancer...) suggesting that the enzyme could have a role in the establishment or progression of these cancers [16, 17, 18, 19, 20]. Moreover, SETMAR has been described as mediating resistance to Topoisomerase II inhibitors in breast cancer cells [17]. To reconcile observations that may appear contradictory (genome integrity *versus* genome instability), we hypothesized that, in physiological conditions, *SETMAR* is expressed at a low level, and then plays a role in maintaining genome integrity. In pathological conditions, *SETMAR* could be over-expressed and increases genetic instability, allowing the cell to bypass cell cycle checkpoints in the presence of damaged DNA.

We focused our studies on SETMAR (de)regulation in glioblastoma multiform (GBM). This model is known for its characteristic chemo- and radio-resistance. GBM is the most aggressive diffuse glioma and the most frequent brain malignancy. Its annual incidence represents 45.2% of all brain and central nervous system malignancy [21]. One of the most important hallmarks of GBMs is tumor heterogeneity: they may contain various cell types, hence the name multiform, the most common being astrocytes. In addition to this cellular heterogeneity, GBMs also contain different morphological zones: central regions are almost entirely necrotic, with only scattered islands of viable neoplastic tissue, mostly around blood vessels. The central necrosis is surrounded by densely cellular tumor tissue consisting of highly anaplastic cells. The third zone is made of healthy tissue infiltrated by tumor cells, which makes the full resection quite impossible [22]. Despite its frequency, GBM remains incurable with a median survival of 3 months if untreated and of 15 months when maximal surgical resection is followed by concomitant radiation therapy and chemotherapy [23]. Recently isolated, GBM stem-like cells are thought to represent the population of tumorigenic cells responsible for GBM resistance and recurrence following surgery and chemotherapy [24]. Biomolecular markers, especially MGMT methylation status and IDH-1 mutations [25, 26] are indicators of prognosis, and of response to chemotherapy and radiotherapy. However, novel therapies that target these mutations are still inconclusive in adapting a therapeutic strategy at the individual level. Identifying new markers and/or therapeutic targets for improving the therapeutic arsenal against GBMs is thus both useful and necessary. For its known biological activities [27], SETMAR must be studied as a potential candidate in this context.

Despite the fact that several transcripts of the *SETMAR* gene have been observed [16], only a single protein of 671 amino acids, firstly described by Lee et al [3], has been studied to date. In the present report, we have focused our attention on the various endogenous *SETMAR* mRNA(s) and protein(s) detected in GBMs from surgical resections. We have demonstrated that a small SETMAR protein, corresponding to a variant that contained mostly the transposase (MAR) domain, was enriched when compared to the full-size 671-amino acids enzyme. The abundance of the small variant could be related to its exceptional stability. We have found evidence that SETMAR expression is not only regulated by alternative splicing, but also by the use of an alternative ATG initiation codon, leading to a 684-amino acids enzyme, in agreement with the recent updating of the NCBI reference sequence NP_006506. Four SETMAR proteins may thus be synthesized in human glioblastomas, each having potentially different biochemical properties and characteristics. The possible role of these various SETMAR enzymes in GBMs biogenesis is discussed.

## RESULTS

### *SETMAR* expression in healthy brain and in 8MGBA cells

**Figure 1:**
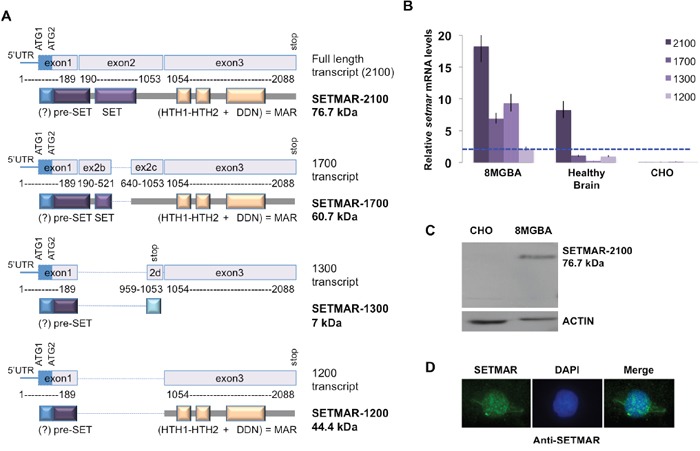
*SETMAR* mRNAs in 8MGBA cells **A**. SETMAR isoforms (mRNA and proteins). Numbering of base pairs was performed according to the sequence presented in 2A. The putative ATG (1 and 2) and the stop codons are indicated. The transcribed region coding the putative α-peptide is represented as a blue box. The expected translated products are drawn below each transcript. mRNA and protein names are given in the right margin. The given molecular weights are the one expected in the absence of α-peptide. The main protein features are represented as colored and annotated rectangles. Spliced regions are represented as blue dotted lines. The question mark (?) indicates the putative α-peptide. HTH: helix-turn-helix motifs; DDN: SETMAR catalytic triad. The transposase (MAR) domain is encoded by exon 3. *SETMAR* 2100 (NM_006515) corresponds to variant 1 [16]; *SETMAR* 1700 (NM_01243723.1) corresponds to variant 2 [16]; *SETMAR* 1300 is very similar to variant B described in [16] and *SETMAR* 1200 corresponds to variant A described in [16]. **B**. Relative *SETMAR* mRNA variants expression in 8MGBA, healthy brain and CHO. The relative quantity for each mRNA was generated from triplicate RTqPCR reactions following normalization to GAPDH. Ct differences between the 1200-mRNA (the less expressed in 8MGBA, taken as the reference, dotted blue line) and other transcripts were calculated. The fold difference for each mRNA was calculated using ΔΔCt method. Bars are medians ± SD. **C**. Western blot analysis of 8MGBA and CHO crude extracts using an anti-SETMAR antibody (top panel). ACTIN was used as control (bottom panel). The detected proteins are indicated in the right margin, with their respective expected molecular weight. **D**. Immuno-localization of endogenous SETMAR-2100. 8MGBA were labeled with anti-SETMAR antibody directed against the pre-SET domain (green signal).

Preliminary characterization of *SETMAR* mRNA in 8MGBA and healthy brain cells was achieved by northern blot experiments and indicated that at least four *SETMAR* mRNA were detected in both cell types (Supplementary Figure 1). Main *SETMAR* cDNAs were cloned and sequenced from 8MGBA, revealing that they were similar to those published during the course of our study [16]: *SETMAR* 2100 was coding for the usual protein, responsible for the biological activities described in the literature; *SETMAR* 1700 was coding for a protein that lacked a part of the SET domain; *SETMAR* 1300 contained a reading frame with a premature stop codon and *SETMAR* 1200 encoded a protein containing mainly the MAR domain (Figure 1A). *SETMAR* expression was quantified by RTqPCR for each mRNA in both 8MGBA and healthy brain. Since *SETMAR* was known as a primate specific gene, no signal was expected from CHO, our negative control (Figure 1B). Our data indicated that the full-length transcript was always the most expressed. *SETMAR* expression was increased in 8MGBA for all transcripts (Figure 1B), the effect being less intense for the 1200-transcript. We have used an antibody directed against exon 1 (shared by all SETMAR putative translated products) to verify which protein variant could be detected in 8MGBA. The specificity of this commercial antibody in western blot was verified since it did not detect any protein in non-primate cells (illustrated by CHO in Figure 1C). Western blot assays demonstrated that SETMAR-2100 was the most abundant isoform in 8MGBA (Figure 1C), whereas SETMAR-1700, SETMAR-1300 and SETMAR-1200 were not detected. As mentioned before, the presence of SETMAR-1300 was not expected because the 1300-mRNA contained a premature stop-codon. The low levels of 1700- and 1200-mRNA accounted for the non-detection of the corresponding proteins.

No detail was available in the literature about the cellular localization of the endogenous native isoform. It has been shown that a recombinant SETMAR-2100 formed nuclear foci at DNA double strand breaks in damaged cells [10] and accumulated in the compact chromatin during the G2/M phase, whereas the overall cellular level did not vary during the whole cycle [6]. The localization of the endogenous SETMAR-2100 was assayed by immunofluorescence (IF) in 8MGBA using an anti-SETMAR antibody. In asynchronous and untreated cells, IF signals indicated that SETMAR-2100 was mainly found in the nucleus (Figure 1D).

### An alternative ATG for SETMAR translation initiation?

**Figure 2:**
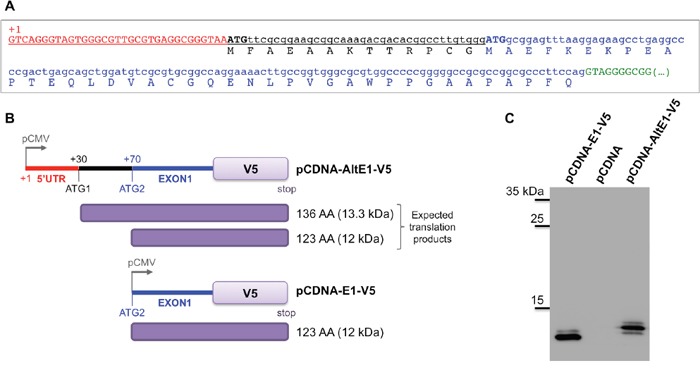
Alternative ATG for SETMAR translation initiation **A**. The sequence of the 5′UTR and of the first exon were obtained by 5′ RACE-PCR. The transcription starting site (TSS) is indicated (+1), 5′UTR in red, ATG1 and ATG2 in capital bold letters, black and blue respectively. The usual exon 1 is in blue, the putative N-terminal peptide of 13 AA (α-peptide) in black, the beginning of the first intron in green. **B**. Schematic view of the vectors used. pCDNA-AltE1-V5 contained the whole 5′ region of *SETMAR* mRNAs, with both ATG; ATG1 was the alternative ATG tested in the assay, ATG2 was the one usually considered as the initiation codon. pCDNA-E1-V5 only contained the usual exon 1, beginning at ATG2. pCMV: CMV promoter. The tag-V5 was in frame at the C-terminal part of exon 1 in both constructs. Expected translation products were indicated in the right margin: 136 AA was the expected product if translation begins at ATG1, 123 AA was the expected product if translation begins at ATG2. **C**. Western blot analysis of crude extracts from 8MGBA transfected by pCDNA-AltE1-V5 or pCDNA-E1-V5, and separated onto a 20% acrylamide gel. A control was performed with pCDNA empty vector. Bands were visualized using anti-V5 antibody. Molecular weight markers are reported on the left margin (in kDa).

To address a missing data, we experimentally defined the *SETMAR* transcription start site (TSS) and we demonstrated that the 5′UTR of *SETMAR* mRNA was quite small (about 70 pb) and contained a first ATG (ATG1) in frame with that viewed as the commonly used for translation initiation (ATG2) (Figure 2A). Depending on the ATG used, SETMAR could have a short N-terminal peptide of 13 amino acids (herein called α-peptide) whose function and/or occurrence has never been described, as the “founder works” [3, 14] had used the 671-amino acids recombinant enzyme beginning at ATG2, on the basis of conventional Kozak sequences found around this downstream ATG. In the absence of specific antibodies, we designed an assay to check whether ATG1 could be used *in vivo* as an alternative codon for the initiation of translation. Two plasmids were constructed. The first (pCDNA-AltE1-V5) contained the whole *SETMAR* 5′ region (from TSS to the end of exon 1, i.e. with ATG1 and ATG2) in frame with the V5-tag. The second (pCDNA-E1-V5) contained the previously studied exon 1 (only containning ATG2) in frame with the V5-tag (Figure 2B). pCDNA-E1-V5 was expected to give a translation product of 123 amino-acids (12 kDa) while pCDNA-AltE1-V5 could generate both a 136 amino-acids (13.3 kDa) translation product if ATG1 was used, and/or a 123 amino-acids (12 kDa) if ATG2 was used. Both plasmids were transfected into 8MGBA. The peptide detected in western blot from cells transfected with pCDNA-AltE1-V5 was larger than that from cells transfected with pCDNA-E1-V5, in agreement with the expected 13 amino-acids size difference (Figure 2C). Our results clearly showed that ATG1 was preferentially used to initiate translation from the mRNA encoded by pCDNA-AltE1-V5, *i.e*. from a messenger containing ATG1 and ATG2. Because the SETMAR endogenous mRNA contained both ATG, it seems reasonable to consider that the endogenous enzyme may start at ATG1, and would thus present the α-peptide at its Nter-end. The putative role of this peptide will be described further.

### *SETMAR* mRNAs and proteins stability

**Figure 3:**
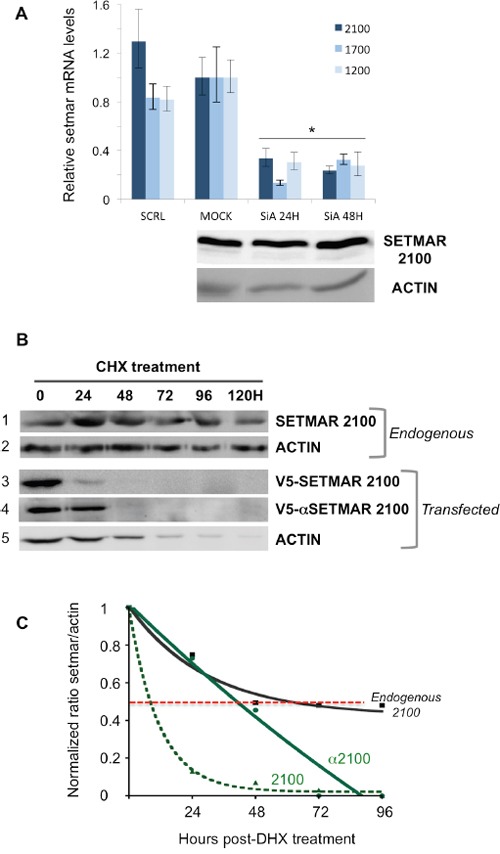
Stability of *SETMAR* products **A**. siRNA assays. Cells were transfected with siRNA directed against exon 3 in order to silence all *SETMAR* mRNA. Top panel: The relative quantity for each mRNA was generated from triplicate RTqPCR reactions following normalization to GAPDH. Ct differences between the 2100-mRNA, 1700-mRNA or 1200-mRNA in 8MGBA (taken as references) and other transcripts were calculated. The fold difference for each mRNA was calculated using ΔΔCt method. Bars are medians ± SD. SCRL: 8MGBA transfected with a scramble siRNA and collected 48H post-transfection. MOCK, non-transfected 8MGBA. SiA: 8MGBA transfected with a specific *SETMAR* siRNA and collected 24 or 48H post-transfection, as specified. Bottom panel: Western blot analysis of crude extracts after the cells were treated as described for the top panel. Bands were visualized using an anti-SETMAR antibody. The detected proteins are indicated in the left. ACTIN was used as a control. **B**. Western blot analyses of crude extracts after the cells were treated by CHX. Bands were visualized using an anti-SETMAR antibody (for the endogenous protein in 8MGBA, track 1) or an anti-V5 antibody for recombinant proteins transfected in 8MGBA (tracks 3 and 4). ACTIN was used as a standard (tracks 2 and 5). **C**. Quantification of SETMAR proteins half-life. Signals recovered in (B) were quantified. For each point, ratios of SETMAR on ACTIN signals were reported as a function of time. Half-lives correspond to the time needed to lose half of the original amount (dotted red line). Black line: endogenous SETMAR; straight green line: V5-α2100-SETMAR; dashed green line: V5-2100-SETMAR.

The stability of *SETMAR* endogenous products was investigated in 8MGBA using siRNA directed against *SETMAR* exon 3, a sequence that should silence all *SETMAR* mRNAs. We observed that the mRNA levels were decreased about 5 to 10 fold 24H post-transfection, this level remaining low 48H post-transfection (Figure 3A, top panel). In contrast, the protein SETMAR-2100 was still present at an unmodified level 48H-post transfection (Figure 3A, bottom panel). The half-life of the endogenous SETMAR-2100 isoform was then determined under Cycloheximide (CHX) treatment. Whereas many proteins in living human cancer cells are known to have half-lives ranging from 45 min to 22.5H in average [28, 29], SETMAR-2100 half-live was amazingly longer, about 60-65H, the protein being still detected 120H post-treatment (Figure 3B-3C). Various hypotheses may account for the unusual stability of SETMAR-2100. Among them, the occurrence of a “pro-like enzyme” involving the α-peptide was considered. To tackle this issue, the half-life of the recombinant V5-SETMAR-2100 and V5-αSETMAR-2100 was compared. 48H post-transfection, protein translation was inhibited by the addition of CHX, and cells were harvested every 24H for western blot analysis using an anti-V5 antibody (Figure 3B-3C). The presence of the V5-tag in our constructs prevented cross-signals with endogenous SETMAR. The double treatment suffered by the cells (transfection plus CHX treatment) resulted in an increased cell death (Figure 3A-3B: compared the ACTIN lanes between non-transfected and transfected cells). Beyond the cell death-related to the experimental conditions, a clear difference was obtained between the two transfected proteins. While the recombinant V5-SETMAR-2100 displayed a half-life of about 10-12H, the recombinant V5-αSETMAR-2100 was more stable, with a half-life of about 40H, suggesting a stabilizing effect of the α-peptide.

### *SETMAR* expression in brain tumors

**Figure 4:**
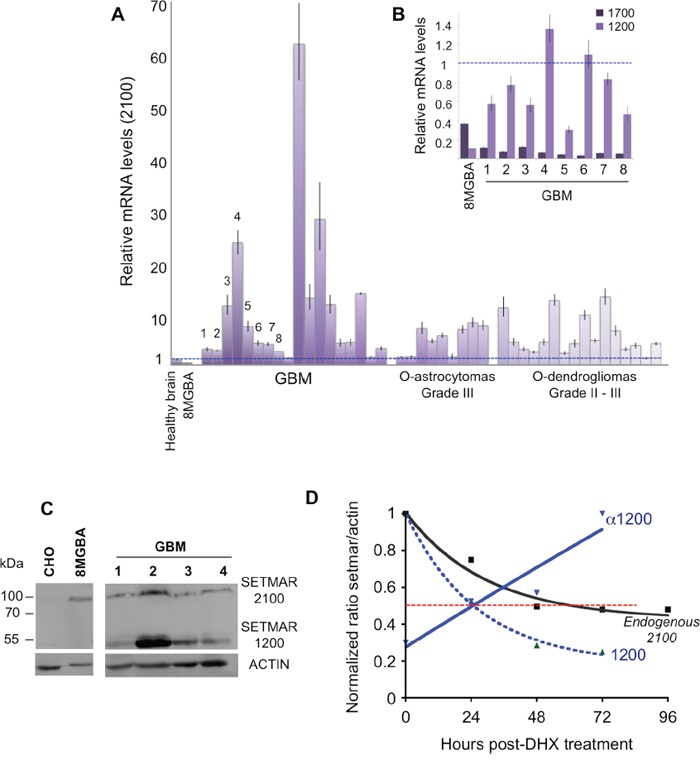
*SETMAR* products in brain tumors **A**. Relative *SETMAR* 2100-mRNA expression in healthy brain, 8MGBA, GBMs (18 samples), oligoastrocytomas (9 samples) and oligodendrogliomas (16 samples). The relative quantity for each mRNA was generated from triplicate RTqPCR reactions following normalization to GAPDH. Ct differences between the 2100-mRNA in 8MGBA (taken as the reference) and other transcripts were calculated. The fold difference for each mRNA was calculated using ΔΔCt method. Bars are medians ± SD. **B**. Relative *SETMAR* 1200-mRNA and 1700-mRNA expression in 8MGBA and GBMs 1 to 8. The relative quantity for each mRNA was generated from triplicate RTqPCR reactions following normalization to GAPDH. Ct differences between the 2100-mRNA in 8MGBA (taken as the reference, dotted blue line) and other transcripts were calculated. The fold difference for each mRNA was calculated using ΔΔCt method. Bars are medians ± SD. **C**. Western blot analysis of CHO, 8MGBA and GBMs 1 to 4 crude extracts using an anti-SETMAR antibody (top panel). ACTIN was used as control (bottom panel). The detected proteins are indicated in the right margin. **D**. Quantification of SETMAR proteins half-life. Signals recovered by western blot (not shown) were quantified. For each point, ratios of SETMAR on ACTIN signals were reported as a function of time. Dotted red line: half-lives. Black line: endogenous SETMAR; straight blue line: V5-α1200-SETMAR; dashed blue line: V5-1200-SETMAR.

We verified whether the over-expression of SETMAR in 8MGBA was a property shared by brain tumors. 43 tissue samples from surgical resections were analyzed by RTqPCR to quantify the 2100-*SETMAR* mRNA. Among them, 16 were grade II or III oligodendroglioma, 9 were grade III oligoastrocytoma, and 18 were *de novo* glioblastoma (GBM). 2100-*SETMAR* mRNA level in 8MGBA was taken as a reference. As shown in Figure 4A, all tumors have 2100-*SETMAR* mRNA levels higher than that of the 8MGBA and consequently than that of the healthy brain. Remarkably, the level of 2100-*SETMAR* mRNA varied considerably from one sample to another, as a picture of the tumors heterogeneities. Tumors that have the apparent highest rates of 2100-*SETMAR* mRNA were GBMs. We then focused our attention on eight samples (GBM 1-8) in which the available samples allowed designing further experiments. The levels of 1200-*SETMAR* mRNA varied in parallel to the 2100-mRNA levels in the eight GBMs when compared to 8MGBA (Figure 4B) and healthy brain (Figure 1B). In contrast, 1700-mRNA levels were systematically lower in GBMs when compared to 8MGBA, remaining slightly higher or similar to that of healthy brain. Western blot analyses from four GBMs (1-4, for which the remaining samples allowed designing further experiments) showed that the proteins SETMAR-2100 and SETMAR-1200 were detected in all GBM, whereas SETMAR-1700 was not, consistently with the low level of 1700-*SETMAR* mRNA (Figure 4C). Surprisingly, SETMAR-1200 was detected as the main SETMAR product in three GBMs among the four analyzed, and regardless to the relative levels of 1200-mRNA *versus* 2100-mRNA (Table 1). We previously observed that the endogenous SETMAR-2100 detected in 8MGBA was surprisingly stable. As it was impossible to directly access to the stability of the GBM endogenous SETMAR, the half-life of recombinant isoforms (V5-SETMAR-1200 and V5-αSETMAR-1200) was analyzed in 8MGBA, and compared to the endogenous 2100 protein found in non-transfected 8MGBA. Our results indicated that in the absence of the α-peptide, SETMAR-1200 displayed a half-life of about 24H (Figure 4D) that was more than the transfected SETMAR-2100, but less than the endogenous SETMAR-2100. In the presence of the α-peptide, αSETMAR-1200 displayed a half-life greater than that of the endogenous SETMAR-2100. The curve representing the half-life of αSETMAR-1200 can appear aberrant, but it merely reflected that the presence of the α-peptide made SETMAR more stable than ACTIN (Figure 4D). As a conclusion, the transfected SETMAR-1200 appeared more stable than the transfected SETMAR-2100 and the α-peptide greatly increased the stability of both variants.

**Table 1:** SETMAR products relative quantification in GBM 1 to 4 and in 8MGBA

	8MGBA	GBM1	GBM2	GBM3	GBM4
% mRNA-2100	91	81	77	96	95
% mRNA-1200	9	19	**33**	**4**	**5**
% SETMAR-2100	100	65	15	35	45
% SETMAR-1200	ND	35	**85**	**65**	**55**

For each data, the relative amount of *SETMAR* mRNA and proteins (from Figure 4) was quantified and given as percentages of 2100 *versus* 1200. ND: non detectable.

**Figure 5:**
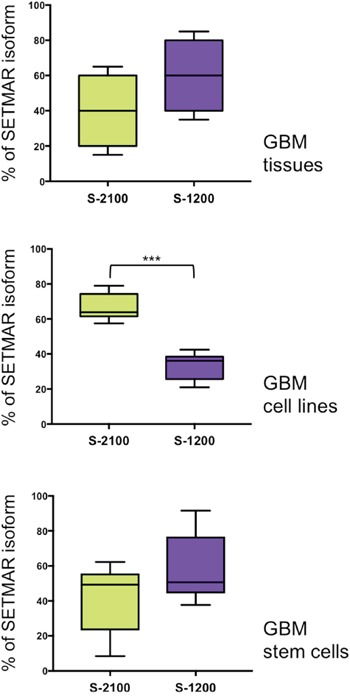
Percentages of each SETMAR protein (2100 *versus* 1200) in three cell “types” Crude protein samples were prepared from GBM tissues (top panel, n=4), GBM derived cell lines, including 8MGBA (middle panel, n=7) and GBM stem cells (bottom panel, n=6). Western blots were analyzed using anti-SETMAR antibodies and quantified. Results were presented as box-plots to compare the relative amount of SETMAR-2100 (S-2100, green boxes) and SETMAR-1200 (S-1200, violet boxes). Wilcoxon matched-pairs signed rank test were performed to test whether differences were significant or not (*** p=0.0002). Note that the top panel was quantified from data presented in Figure 4C and Table 1.

Proteins analyses revealed a major difference between GBMs tumor tissue and a GBM derived cell line, 8MGBA: the presence of higher levels of SETMAR-1200 in GBMs. The faint level of SETMAR-1200 in GBM derived cell lines was verified by analyzing six other lines (U-87 MG, T98G, CRL 2020, A172, Hs 683 and 42MGBA). Western blots were performed (Supplementary Figure 3) and quantified as in Figure 4C, and results were reported as box-plots to compare the overall enrichment in SETMAR-1200 *versus* SETMAR-2100 in various sample categories. Results are shown in Figure 5. In GBMs, the statistical analysis revealed no significant differences between the amounts of SETMAR-1200 *versus* SETMAR-2100, despite a trend towards SETMAR-1200 (Figure 5, top panel). The GBM derived cell lines displayed a pattern dramatically different, with a strong prevalence of SETMAR-2100 (Figure 5, middle panel). By confirming the results observed for 8MGBA, these results, showing a consistent difference between GBMs tumor tissue and GBM derived cell lines, suggested that the amount of each SETMAR proteins (2100 *versus* 1200) may vary according to parameters that remained to be defined. Hypothetically, tumor cells may contain various levels of SETMAR-1200 (from high to faint), and/or various levels of SETMAR-2100, resulting in the median outcome observed for GBMs. This hypothesis was sustained by the fact that GBMs are known as highly heterogen, and organized into territories with various cell phenotypes [30]. In contrast, GBM derived cell lines were homogenous within each line, and shared a same trait, mainly producing the large SETMAR-2100. This result led us to look for GBM cells that may produce over amount of SETMAR-1200. We therefore verified whether GBM stem cells satisfy this hypothesis. We used six GBM stem cells isolated and characterized at the CHU of Poitiers [31] to perform western blots as described in Figure 4C. After quantification, results were reported as box-plots to compare the enrichment in SETMAR-1200 *versus* SETMAR-2100 (Figure 5, bottom panel). The statistical analysis indicated that the short SETMAR-1200 was more abundant in GBM stem cells than the larger SETMAR-2100, giving to GBM stem cells a profile different from that of cancer cell lines.

### SETMAR-1200 NHEJ-properties

**Figure 6:**
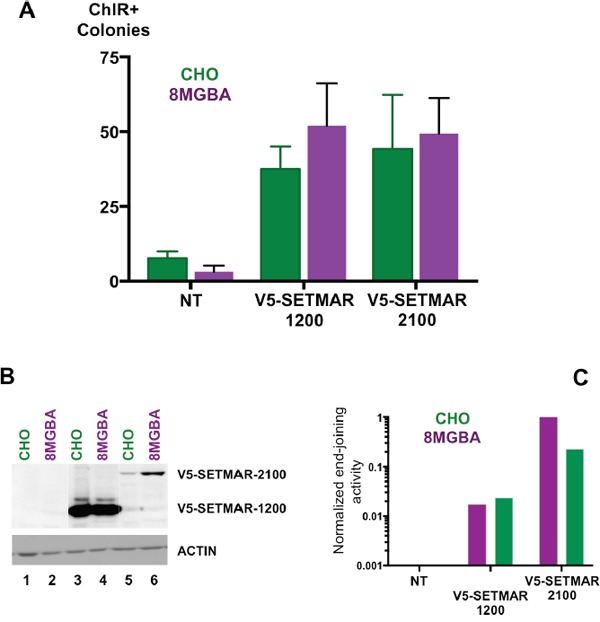
End-joining assays **A**. *In vitro* DNA end-joining in cell-free extracts from CHO (green), and 8MGBA (purple) transiently expressing V5-SETMAR-1200 or V5-SETMAR-2100 as specified. NT: assays performed with cell extracts from non-transfected cells. Nuclear extracts were incubated with a linearized plasmid DNA before transformation into *E. coli* for colony counts. Bars are the mean (± SD) of at least 4 independent assays done with two different cell-extract preparations. **B**. Western blot analysis of cell extracts used in A/. NT: nuclear extracts from non-transfected cells, V5-SETMAR-1200: nuclear extracts from cells transiently expressing V5-SETMAR-1200, V5-SETMAR-2100: nuclear extracts from cells transiently expressing V5-SETMAR-2100. Bands were visualized using anti-V5 antibody. The detected proteins are indicated in the right margin. ACTIN was used as a control. **C**. Normalized end-joining activities. For each condition [CHO (green) and 8MGBA (purple)], the number of colonies obtained in A was adjusted for the amount of SETMAR isoform (V5-1200 and V5-2100) detected in B. The smallest value was taken as reference.

We reported for the first time the occurrence in GBMs and GBM stem cells of a SETMAR isoform never described and consisting essentially of the MAR domain: SETMAR-1200. The study of the transposase-like activities of the MAR domain of SETMAR has brought evidences that the so-called “EXON3” protein retained robust transposase activities despite a severe defect for cleavage at the 3′ end of *mariner* elements [14]. In contrast, HsMAR-RA, the ancestor of SETMAR transposase, displayed a fully operational transposase activity [15]. It seemed thus important to verify whether SETMAR-1200 retained the ability to repair DNA double strand breaks. As a starting point to address this issue, we used an intra-molecular end-joining assay that measured joining of linearized plasmid DNA in a cell-free system. We showed that both proteins (V5-SETMAR-2100 and V5-SETMAR-1200) were able to promote DNA joining reactions (Figure 6A). Control experiments (performed with non-transfected 8MGBA and CHO nuclear extracts) showed a weak signal corresponding to background; we therefore concluded that the positive signal obtained with transfected cells was only due to V5-SETMAR-2100 and V5-SETMAR-1200 proteins. In addition, analyses of the repaired plasmids indicated that a precise repair of the compatible ends was done, since the *BamH1* site was always restored (not shown). Those data indicated that the MAR domain of SETMAR was necessary and sufficient to promote DNA end-joining. The difference between the two proteins led us to measure the amount of each protein in the extracts used for the assay (Figure 6B). V5-SETMAR-1200 was at least 10-times more abundant than V5-SETMAR-2100. If it was legitimate to estimate a specific DNA end-joining activity by evaluating the ratio of activity over protein amount, one could propose that V5-SETMAR-1200 is less efficient than V5-SETMAR-2100. While SETMAR-1200 isoform has kept at least some of the DNA repair properties of the full-length enzyme, its lower efficiency suggests a main role of the SET domain in DNA repair activity.

## DISCUSSION

SETMAR is a recently characterized fusion protein comprising a SET histone methylase domain and a transposase domain, present only in anthropoid primates. It is a DNA repair component that participates in non-homologous end-joining [9]. SETMAR has retained all the activities of the *mariner* transposase, excepted the DNA second strand cleavage [14]. In addition to its well characterized functions (see Introduction), SETMAR is an effector of CHK1 [11, 32], an activity that may interfere with the normal cell cycle. All these properties suggest that SETMAR could play a role in various oncogenic processes. However, it is striking that the expression pattern of the endogenous SETMAR proteins, and especially which isoforms were expressed in cancer cells, had never been studied. We focus our interest on SETMAR expression in GBM, a tumor model known for its resistance to both radio and chemotherapy, a characteristic that is *a priori* coherent with the known biological activities of SETMAR.

Our main results were: (1) a lack of correlation between SETMAR mRNA and protein levels, while the rate of mRNA was routinely used as an indicator of SETMAR expression [16]. (2) The presence of an alternative start codon (ATG1) which was the preferred for initiating translation in 8MGBA. The SETMAR protein(s) containing the resulting additional 13-amino acids N-terminal peptide displayed an increased stability. (3) The presence in GBM primary tumors and GBM stem cells of over-expressed endogenous SETMAR, suggesting a possible role in the natural history of these tumors. Two SETMAR variants were detected: the already characterized SETMAR-2100, and more interestingly, SETMAR-1200, a variant mostly constituted of the transposase domain. (4) The MAR domain contributed to the DNA end-joining activities exerted by the full-length protein.

Our results suggest a sophisticated control of the regulation of *SETMAR* gene expression, acting both at the transcriptional and post-transcriptional levels. The probable protein stabilization *via* the α-peptide suggests that dilution over cell division is the main cause of SETMAR disappearance [28]. The presence of N-terminal α-peptide has been correlated to an increased protein stability in other systems, mainly by inducing conformational changes [33, 34]. The SET domain of SETMAR displays the usual conformation of Histone-lysine N-methyltransferase (PDB ID: 3BO5): a pre-SET domain, a core SET domain and a post-SET domain. The pre-SET structural region contains an eponymous Zn-cluster that characterizes this class of pre-SET domain and provides structural stabilization [35]. In the crystal structure of the protein (PDB ID: 3BO5), the α-peptide was present but its structure undetermined. In contrast, the pre-SET domain was completely and correctly structured, providing stabilization of the whole protein. It is tempting to suggest that the lack of the α-peptide interfered with the proper folding of the pre-SET domain and thus could perturbe the whole protein organization. Finally, the α-peptide did not appear to be a signal peptide that would modify SETMAR cellular distribution (Supplementary Figure 2). We provided evidence that ATG1 was the preferred translation initiation site in 8MGBA, and that endogenous SETMAR were very stable proteins. Taken together, these data allowed us to propose that endogenous SETMAR proteins can contain the α-peptide in their N-terminal part, at least at some stages of GBM biogenesis.

The high stability of the α-SETMAR protein is rather counter-intuitive, as transcription factors, signal and cell-cycle-specific proteins, and chromatin modifying enzymes are usually unstable proteins, with short half-life fitting the highly dynamic processes that they regulate [29]. We would like to propose, as an attractive working hypothesis, that four SETMAR proteins can (co)-exist in the various GBM cells and can contribute to GBMs biogenesis. SETMAR-2100 would be the “usual” variant. It is detected at low levels in many differentiated cells and plays a protective role against double-strand breaks. The increased amount of SETMAR-2100 detected in various cancer cells could be due to an enhanced stability, that we associate to a translation initiation change from ATG2 to ATG1. Abnormal amounts of SETMAR-2100 would begin to deregulate the normal SETMAR function, allowing the cell to achieve its cycle, even in presence of damaged or miss-repaired DNA [17, 18]. It has been shown recently that SETMAR-2100 methylates snRNP70 at K130 [5]. Strikingly, snRNP70 is a key early regulator of 5′ splice site selection, and its methylation by SETMAR could thus participate in the regulation of its own alternative splicing, allowing the production of SETMAR-1200 (containing or not the α-peptide). Our results suggest that these events, if they occur, could arise very early in tumorigenesis since GBM stem cells clearly contain more SETMAR-1200 than SETMAR-2100. Currently, it is not possible to know whether endogenous SETMAR-1200 contains or not the α-peptide. The increased amount of this variant in GBM stem cells may be due to an increased translation efficiency from ATG2 (due to intrinsic properties of this shorter mRNA), to a translation initiation from ATG1, or to a combination of both mechanisms. In addition, the physiological functions of SETMAR-1200 remain to be fully understood. We have shown that it is less efficient than SETMAR-2100 in DNA repair by NHEJ. This sole property may worsen the state of the cell. Because SETMAR proteins act as dimers through their transposase domain [36, 37], SETMAR-1200 could poison SETMAR-2100 functional units, or act as a direct competitor of SETMAR-2100 partners that do not involve the SET domain. Various examples of transposase-transposase interactions and/or of dominant negative complementation were previously published for transposases of the same family [38, 39, 40, 41]. The status of SETMAR-1200 should be assessed in GBMs on large patient cohorts. Moreover, the high level of SETMAR-1200 in GBMs and GBM stem cells, associated to its role in end-joining DNA repair, makes it a new potential therapeutic target to investigate, in addition to the large protein already considered by others [17]. For instance, the existence of anti-SETMAR inhibitors [27] may offer the opportunity to test the involvement of various SETMAR(s) on cells that have undergone genotoxic damage.

## MATERIALS AND METHODS

### Cells

8MGBA (human GBM, #ACC-432 DSMZ) and CHO (Chinese hamster ovary, #ACC-110 DSMZ) cells were cultured as recommended by the Liebniz Institute DSMZ German Collection of Microorganisms and Cell Cultures. 8MGBA were transfected with FugeneHD (Promega). CHO were transfected with jetPEI (Polyplus transfection). Proteins translation was inhibited by the addition of 10μg/ml CHX on 80%-confluent cultures, and cells were harvested every 24H for western blot analysis.

U-87 MG, T98G, CRL 2020, A172 and Hs683 cell lines were cultured as recommended by ATCC, and 42MGBA was cultured as recommended by the Liebniz Institute DSMZ German Collection of Microorganisms and Cell Cultures. All were obtained from human glioblastoma, except Hs683, obtained from a glioma.

Six GBM initiating stem cells (named CSG-1 to 3 and CGS-9 to 11), isolated in the Laboratoire de Cancérologie Biologique (CHU de Poitiers, France) were cultured as neurospheres as previously described [31, 42].

### Clinical samples

Histologically confirmed GBMs (grade IV GBM #1 to 8, according to the OMS nomenclature) were taken from the material of surgical resection during the course of standard diagnosis procedure (CHRU of Tours). RNA from histologically confirmed brain tumors (Biobank N° DC-2012-1584) were taken from the material of surgical resection during the course of standard diagnosis procedure (grade IV GBM #10 to 19, grade II or III oligodendrogliomas, and grade III oligoastrocytomas, according to the OMS nomenclature) came from the CHRU of Clermont-Ferrand. All patients (from Tours and Clermont-Ferrand) gave informed consent prior to collection of specimens according to institutional guidelines. From Clermont-Ferrand, glioma samples resected between 2007 and 2014 were obtained from the Neurosurgery Unit at the Clermont-Ferrand University Hospital Center, France (“Tumorothèque Auvergne Gliomes”, ethical approval DC-2012-1584). From Tours, GBM samples were resected between 2013 and 2014 and obtained from the Neurosurgery Unit at the Tours University Hospital Center, France (ethical approval CPP of Tours, Philippe Bertrand, 2013).

### Plasmids

V5-SETMAR expression plasmids were constructed using the pCDNA3.1(+)(Invitrogen) as the backbone. The V5-tag was first cloned at the *NheI*/*HindIII* restriction sites, giving pCDNA-V5. The latter was then used to clone at the *HindIII*/*XhoI* restriction sites *setmar* cDNA isoforms (2100 and 1200) in frame with the V5-tag, giving two constructs: pCDNA-V5-SETMAR2100 and pCDNA-V5-SETMAR1200. In these constructs the α-peptide was missing as well as the SETMAR usual ATG, so that the only used ATG for translation was that of the V5-tag. pCDNA-V5-αSETMAR2100 and pCDNA-V5-αSETMAR1200 were obtained by cloning the α-peptide in frame between V5 and SETMAR sequences, at the *Xho1* restriction site.

Plasmids for the alternative ATG assays were constructed using the pCDNA3.1(+)(Invitrogen) as the backbone. The V5-tag was first cloned at the *XbaI*/*EcoR1* restriction sites, giving pCDNA-V5-stop, in which the ATG of the V5 ORF has been removed, whereas a stop codon has been added at the end of the V5 sequence. pCDNA-V5-stop was used to clone at the *HindIII*/*XbaI* restriction sites the two versions of *SETMAR* exon 1, giving respectively pCDNA-E1-V5 and pCDNA-AltE1-V5. All constructs were verified by sequencing. For transfections, plasmids were purified using the NucleoBond Xtra Midi Plus EF kit (Macherey Nagel).

### RNA

Total RNAs were extracted from 2×10^6^ cultured cells or 50 mg of tumor tissues (Nucleo spin RNA kit, Macherey Nagel). In the case where poly(A+) RNAs were needed, total RNAs were first extracted from 20×10^6^ cells, and then purified with Nucleo Trap mRNA mini kit (Macherey Nagel). Human brain poly(A+) mRNAs were purchased from Clontech (#636102). It corresponds to normal, human brain (whole) pooled from eight Caucasian males, ages: 43-66; cause of death: sudden death. Because the tumors we analyzed were heterogeneous both between and within samples, we assumed that mRNAs corresponding to whole brain were the best and robust reference. To knock down SETMAR expression, cells were transfected with the si-SETMAR (sense) 5′-AGAACUCAAUGUCAACCAUUCUACG-3′(from Origen). 2×10^6^ cells were transfected using the Lipofectamine RNAi Max (Invitrogen), following the manufacturer's instructions. A SETMAR scrambled siRNA was used as a negative control.

### RTqPCR analyses

Isoform-specific primers were designed as follows: 2100 forward 5′-CAGAGTGGTCCAGAAAGGTC-3′, 2100 reverse 5′-GCATATTCACAGACAAACCTTC-3′, 1700 forward 5′-CAGAGTGGTCCAGAAAGGTC-3′, 1700 reverse 5′-GGGGCAGTACAGAGAACTTC-3′, 1200 forward 5′-GGCGCCCTTCCAGACTA-3′, 1200 reverse 5′-ATGCATTGTTGATGTTGCGAGTT-3′. One microgram of total RNA was used for reverse transcription reactions using the first strand cDNA synthesis kit (Fermentas), with random hexamers. cDNAs were then diluted to a final concentration of 10 ng/μl and amplified using a BioRad Opticon instrument. Amplifications were performed using the Mesa Green qPCR Master SYBR Green I, following the instructions of the manufacturer (Eurogentec). Quantitative data were recovered using the BioRad CFX Manager software. cDNA samples were assayed in triplicates. The *GAPDH* housekeeping gene was used as the endogenous normalizer. RQ was calculated using the conventional method of the ΔΔCt, where RQ (Relative Quantification) = 2^-Δ ΔCt^.

### Proteins

For crude cell extracts, 10^6^ cells were washed in PBS 1x and scraped directly in 250 μl of Laemmli buffer containing 50 mM DTT. For crude tumor extracts, 50 mg of tissues were ground in 500 μl Laemmli buffer containing 50 mM DTT and sonicated. Preparation of nuclear extracts were performed as previously described [43]. The final protein concentration was measured by Bradford quantification (Biorad).

### Antibodies

Endogenous SETMARs (2100 and 1200 isoforms) were detected using a SETMAR rabbit polyclonal antibody (ab-129455, abcam, dilution1/2500), followed by peroxidase-coupled secondary antibody (SantaCruz) and an enhanced chemi-luminescence reaction (ECL signal, GE healthcare), prior to visualization by a CCD camera (Fuji Las 4000). ACTIN was detected using a Beta-Actin chicken polyclonal antibody (ab-13822, abcam, dilution 1/5000). V5-tagged SETMAR proteins were detected using a V5 Mouse Monoclonal Antibody (Invitrogen, dilution 1/5000).

### Immunofluorescence staining

IF were performed as previously described [44]. Endogenous SETMAR was detected using an anti-SETMAR antibody (sc-103211, Santa Cruz, dilution 1/50) and revealed by an anti-goat secondary antibody (dilution 1/100) conjugated to Alexa Fluor 488 dye (Jackson Immuno Research). V5-SETMARs were detected by anti-V5 antibody (ab-129455, abcam, dilution 1/5) revealed by an anti-mouse secondary antibody (dilution 1/100) conjugated to FITC (Invitrogen). Nuclei were stained with DAPI. Cells were imaged using a microscope Nikon Eclipse T*i* and NIS-Elements software. Images were processed using the software ImageJ.

### Transcription start site (TSS)

*SETMAR* TSS was determined using 5′ RACE-PCR. First strand cDNA synthesis was performed using 0.5μg of 8MGBA poly(A+) mRNAs, according to the manufacturer's instructions (first strand cDNA synthesis kit, Fermentas). The primer was directed against SETMAR Exon 1 sequence (5′-CTGGAAGGGCGCCGGCGC-3′). After purification (PCR Clean-up kit with NTC buffer, Macherey-Nagel Inc.), a poly(G) tail was added at the 3′OH end of the neo-synthetized cDNAs, using a Terminal Deoxinucleotidyl Transferase (TdT)(Promega). Poly(G) cDNAs were purified and amplified by PCR with the following primers: forward 5′-CTGGAAGGGCGCCGGCGC-3′, and reverse 5′-C(14)-3′, and settings: 95°C for 30 sec, 62°C for 1 min and 72°C for 30 sec, for 34 cycles. PCR products were cloned into the pGEMT-Easy vector (Promega), before sequencing (MWG Operon). Resulting sequences were aligned against the *SETMAR* gene sequence (NM_006515) to determine the transcription start point.

### End-joining assays

The DNA substrate consisted of pBC (carrying the chloramphenicol resistance gene) (Stratagene) digested once by *BamH1*, and purified by phenol-chloroform extraction. Assays were performed as previously described [8]. Reactions mixtures (100 μl) contained 60 mM potassium acetate, 50 mM Tris-HCL (pH 7.5), 2 mM ATP, 1 mM DTT, 0.5 mM Mg-Acetate, 5 mM MgCl_2_, 2.5 mM dNTPs and 100 μg/mL BSA. Briefly, linear pBC (2.5 μg) were incubated with crude nuclear extracts (20 μg) obtained from cells (8MGBA or CHO as specified) transfected with pCDNA-V5-SETMAR-2100 or pCDNA-V5-SETMAR-1200. Crude nuclear extracts from non-transfected cells were used as control. Repaired (circular) plasmids were then purified by phenol-chloroform extraction and used to transform competent E. *coli* (JM109) for colonies formation on LB-chloramphenicol plates. Using pBC backbone was necessary to prevent false positive results relying on the presence of ampicillin resistant plasmids in crude nuclear extracts obtained from transient transfected cells. Each point was performed in triplicates and repeated with two to three different nuclear extracts preparations. Repaired plasmids were extracted from JM109 colonies and the junctions were sequenced.

## SUPPLEMENTARY FIGURES


