# Supplementary material for: SETMAR isoforms in glioblastoma: A matter of protein stability
Source: Oncotarget. 2016 Dec 25;8(6):9835–48. doi: 10.18632/oncotarget.14218 (PMC5354774; doi:10.18632/oncotarget.14218)
Supplement: Supplementary file 1 [file oncotarget-08-9835-s001.pdf]

SETMAR isoforms in glioblastoma: A matter of protein stability

SUPPLEMENTARY FIGURES

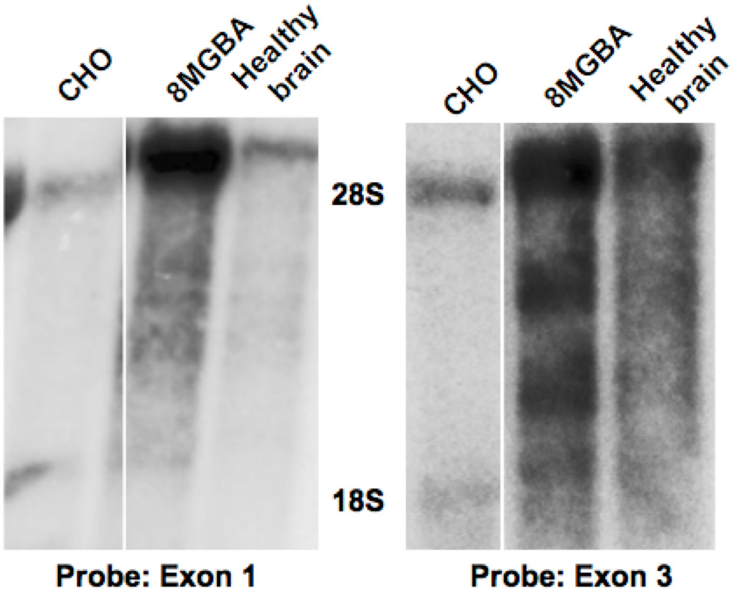

Supplementary Figure 1: Brain *SETMAR* mRNA detected by Northern blot. Briefly, 2-3 µg poly(A+) RNAs were separated on 1% agarose/formaldehyde gel, and transferred to a Hybond XL membrane (Amersham biosciences). RNA probes directed either against SETMAR exon 1 or exon 3 were made using Maxiscript Kit (Invitrogen) and hybridized according to the manufacturer's instructions (NorthernMax kit, Ambion). After washing, the membrane was exposed for 16H on a Phosphor Screen (STORM Molecular Dynamics). Traces of 18S and 28S rRNA were always detected. As expected, no transcript was detected in CHO cells, even with a stronger exposure (not shown). Several specific transcripts were detected in both 8MGBA cells and healthy brain. According to previously published works (see main text) these transcripts resulted from alternative splicing of *SETMAR* pre-mRNA(s).

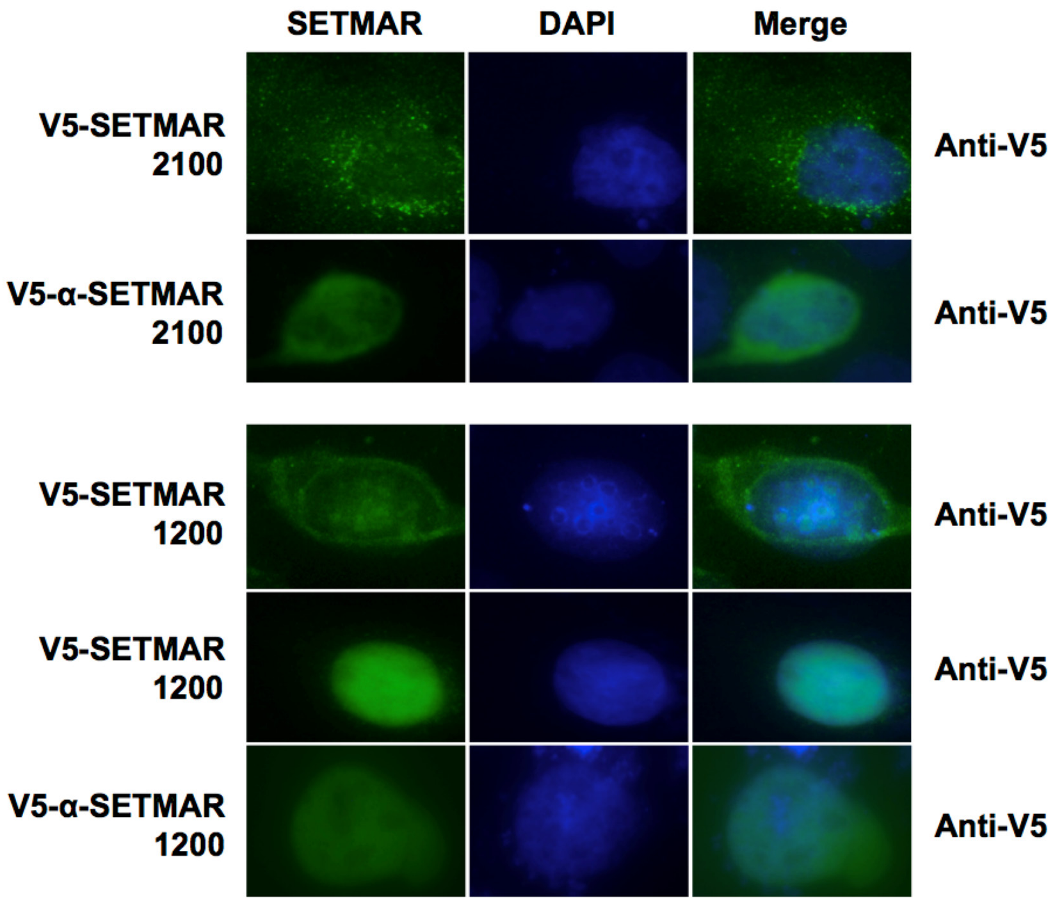

Supplementary Figure 2: SETMAR cellular localization. We have investigated a possible role of the α-peptide in SETMAR cellular location, checking whether SETMAR-1200 and SETMAR-2100 behaved similarly or not, and how the α-peptide influenced they cellular location. The transfected V5-SETMAR-2100 was equally detected in the cytoplasm and in the nucleus, contrary to what had been observed for the endogenous protein (Top panel). Transfection of V5-SETMAR-1200 resulted in two kinds of signals. In most cases, SETMAR-1200 was detected both in the cytoplasm and in the nucleus (similarly to SETMAR-2100, Bottom panel). In some instances (25-30%), a strong nuclear signal was observed, with no labelling of the cytoplasm. We found no differences in cellular location of SETMAR proteins containing or not the α-peptide, whatever the isoform considered. The only difference between SETMAR proteins containing or not the α-peptide was that the signal observed for α-SETMAR was less spotted.

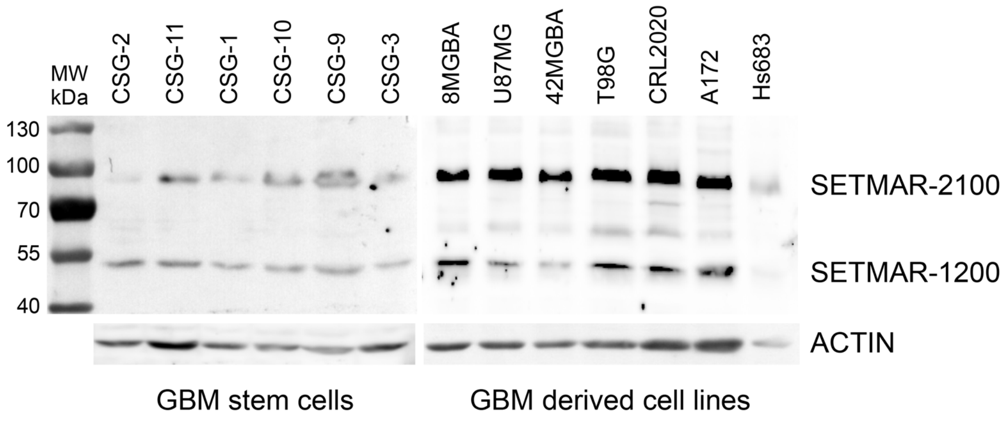

Supplementary Figure 3: SETMAR isoforms detected by Western blot in various GBM cell lines. Here are shown representative Western blots used to quantify SETMAR-2100 and SETMAR-1200 in various cell lines (Figure 5 of the main text).
